# The Pathomechanism and Current Treatments for Chronic Interstitial Cystitis and Bladder Pain Syndrome

**DOI:** 10.3390/biomedicines12092051

**Published:** 2024-09-10

**Authors:** Wan-Ru Yu, Jia-Fong Jhang, Yuan-Hong Jiang, Hann-Chorng Kuo

**Affiliations:** 1 Department of Nursing, Hualien Tzu Chi Hospital, Buddhist Tzu Chi Medical Foundation, Hualien 970, Taiwan; wanzu666@gmail.com; 2 Department of Urology, Hualien Tzu Chi Hospital, Buddhist Tzu Chi Medical Foundation, and Tzu Chi University, Hualien 970, Taiwan; alur1984@hotmail.com (J.-F.J.); redeemerhd@gmail.com (Y.-H.J.); 3 Institute of Medical Science, Tzu Chi University, Hualien 970, Taiwan

**Keywords:** cystitis, urine biomarker, bladder inflammation, bladder pain syndrome

## Abstract

Interstitial cystitis/bladder pain syndrome (IC/BPS) is a chronic and debilitating condition characterized by symptoms such as bladder pain, frequent urination, and nocturia. Pain is typically perceived in the lower abdomen, pelvic floor, or urethra, causing significant discomfort and impacting quality of life. Due to the similarity of its symptoms with those of overactive bladder and acute bacterial cystitis, patients often face misdiagnosis and delayed appropriate treatment. Hunner’s (HIC) and non-Hunner’s IC (NHIC), each with distinct clinical presentations, urothelial dysfunction, chronic inflammation, and central sensitization and thus multimodal symptomatic treatment approaches, may be the most common pathogeneses of IC/BPS. Treatment of IC/BPS should involve identifying the different clinical phenotypes and underlying pathophysiology causing clinical symptoms and developing strategies tailored to the patient’s needs. This review discusses the roles of urine biomarkers, bladder inflammation, and glycosaminoglycans in the pathogenesis of IC/BPS. Various bladder treatment modalities are explored, including glycosaminoglycan replenishment, botulinum toxin A injection, platelet-rich plasma injection, low-energy shock waves, immunosuppression, and low-dose oral prednisolone. Pelvic floor muscle physiotherapy and bladder therapy combined with psychiatric consultation can help alleviate psychological stress and enhance the quality of life of patients with IC/BPS. Elucidating the pathological mechanisms and exploring diverse treatment options would help advance the care of individuals suffering from this challenging bladder condition.

## 1. Introduction

Interstitial cystitis/bladder pain syndrome (IC/BPS) is a chronic and debilitating bladder disease with clinical symptoms of bladder pain, frequent urination, and nocturia. Pain is frequently felt in the lower abdomen, pelvic floor, or urethra. Because the symptoms of IC/BPS are similar to those of overactive bladder or acute bacterial cystitis, patients are often misdiagnosed and/or receive unsuitable treatment [1,2]. Before making a diagnosis of IC/BPS, routine urinalysis and urine culture should be performed to exclude the presence of acute or chronic bacterial cystitis. In addition, for patients who could be misdiagnosed with bladder hypersensitivity, videourodynamic studies might be necessary to exclude detrusor overactivity, bladder outlet obstruction, or neurogenic lower urinary tract dysfunction [3]. A potassium chloride test may also help identify urothelial dysfunction [4]. Patients with IC/BPS may have urgency symptoms but not urinary incontinence. Instead, this urgency is typically directed towards avoiding bladder pain [5].

On cystoscopy, an erythematous patch with radiating vessels called a Hunner’s lesion is usually observed on the posterior wall. Patients with this feature are considered to have Hunner’s IC (HIC) [6]. Patients with IC/BPS symptoms but without a Hunner’s lesion typically present with characteristic glomerulation after cystoscopic hydrodistension under anesthesia and are thus diagnosed with non-Hunner’s IC (NHIC) [7]. Patients with NHIC often have multiple associated organ complaints, including gastroesophageal reflux disease, myofascial pain, insomnia, depression, and anxiety [8]. HIC and NHIC are considered to be two distinct bladder disorders [9]. Although IC/BPS has been recognized for more than 100 years, its underlying pathophysiology has not been fully elucidated. Therefore, treatment of IC/BPS is usually symptomatic [10]. Patients may experience frequent flare-ups of IC/BPS symptoms which significantly affect their quality of life. This article reviews the recent clinical and basic research findings to elucidate the pathophysiology, clinical characteristic findings, and treatment strategies for IC/BPS.

This article narratively reviewed the literature regarding basic and clinical research on the clinical characteristics, cystoscopic features, urine biomarkers, bladder inflammation, and urothelial dysfunction in the pathogenesis of IC/BPS. Relevant articles were obtained by searching the PubMed, MEDLINE, and ScienceDirect databases from 1982 to April 2024 using the keywords ‘interstitial cystitis’ or ‘bladder pain syndrome’ or ‘painful bladder syndrome’ and ‘pathophysiology’ or ‘bladder therapy’. The search results were limited to articles written in English and full-text articles. The literature on various novel bladder treatment modalities developed in recent years included glycosaminoglycan replenishment, botulinum toxin A injections, platelet-rich plasma injections, low-energy shock waves, immunosuppression, and low-dose oral prednisolone, according to the differing underlying pathophysiology of IC/BPS. Since the pathophysiology of IC/BPS is different for individual patients with different clinical phenotypes, the treatment strategy should be personalized. The aim of this review is to provide a rational and effective multimodal treatment strategy for patients with IC/BPS.

## 2. The Pathophysiology of NHIC

Several possible pathogeneses of IC/BPS have been proposed based on patients’ medical history, histopathological bladder findings, urinary biomarker analysis, and immunohistochemical studies. Various conditions may contribute to the development of IC/BPS, including autoimmune reactions after acute bacterial cystitis; mast cell activation due to toxins, stress, or allergies; urothelial dysfunction after bladder wall injury; neurogenic activation through the upregulated release of sensory fibers by neuropeptides; and central sensitization [11,12]. However, none of these possible pathogeneses can encompass all the clinical characteristics and treatment outcomes of IC/BPS. The initial bladder insult may trigger an inflammatory cascade that causes local and central inflammation and the subsequent manifestation of clinical symptoms. The levels of pathophysiology of IC/BPS may begin from urothelial injury caused by acute bacterial infection, intravesical foreign bodies, intravesical instrumentation, or surgical trauma, followed by suburothelial inflammation. If acute inflammation is not resolved, chronic inflammatory cells may infiltrate into the suburothelium and the detrusor muscle, resulting in pan-cystitis and chronic scar formation [8]. The inflammatory reaction may extend into the dorsal horn ganglia and the corresponding sacral cord, resulting in central sensitization and multiple functional somatic symptoms, such as pelvic floor, urethral, or pelvic pain [13]. If patients with IC/BPS are not treated adequately, the inflammatory process may persist at any level or at different levels of pathophysiology, resulting in several different clinical symptoms and characteristic urinary tract findings.

## 3. Urothelial Dysfunction and Treatments for NHIC

**Figure 1:**
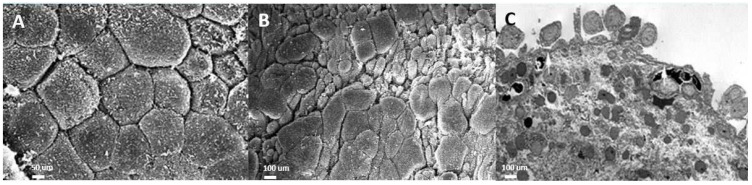
Electron microscopic (EM) examination of the ultrastructure of bladder urothelium in patients with (**A**) a normal urothelium (scanning EM), (**B**) non-Hunner’s interstitial cystitis (IC; scanning EM), and (**C**) Hunner’s IC (transmission EM).

The urothelium in IC/BPS bladders is impaired in isolating urine and the bladder wall, which prevents the influx of urinary acid, potassium, and toxins into the suburothelial space. Chronic inflammation of the urothelium and the bladder wall results in defective apical umbrella cells, incomplete coverage of glycosaminoglycans (GAGs), and the exposure of intermediate cells to urine solutes and potassium [14]. On electron microscopic examination, the layers of the urothelial cells in IC/BPS are eroded or even lost in severe cases [15]. The normal uroplakin plaques covering the normal urothelial cell membrane and the vesicles underneath the cell membrane are missing in IC/BPS urothelial cells, resulting in decreased distensibility of the urothelium during cystoscopic hydrodistension [16] (Figure 1).

**Figure 2:**
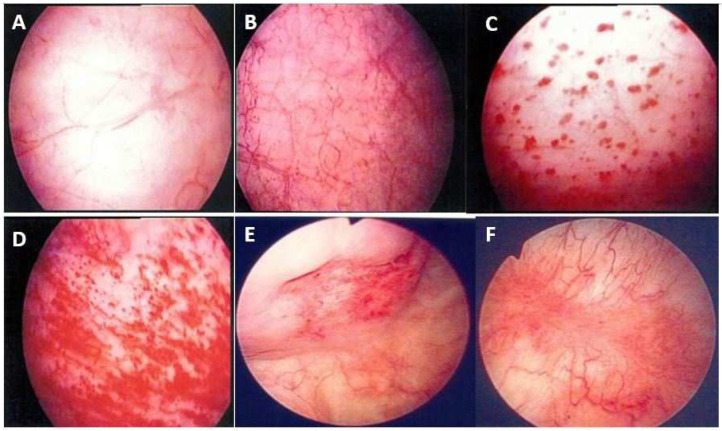
Cystoscopic findings of interstitial cystitis/bladder pain syndrome under hydrodistension: (**A**) normal urothelium, no glomerulation; (**B**) grade 1 glomerulation; (**C**) grade 2 glomerulation; (**D**) grade 3 glomerulation; (**E**) Hunner’s lesion with urothelial crack; and (**F**) Hunner’s lesion with hemorrhagic patch. Magnification ×10.

Therefore, during cystoscopic hydrodistension, the maximal bladder capacity is reduced in IC/BPS bladders, resulting in distention-induced glomerulation hemorrhage. Several different phenotypes emerge after cystoscopic hydrodistension [13]. Glomerulation hemorrhage may be very mild (covering less than two quadrants), mild but diffused (covering more than three quadrants), or severe and diffuse (with splotch hemorrhage or waterfall bleeding) or appear with fissures or cracks [7] (Figure 2).

GAG supplementation may protect the urothelium from an influx of toxins or solutes from the urine, thereby preventing the sensitization of the sensory afferent nerves and further chronic inflammation [17]. The currently available surface protectants include oral pentosan polysulfate and intravesical instillation of heparin, hyaluronic acid, and chondroitin [10,18]. Although data from large randomized controlled studies are limited, long-term clinical observations and anecdotal experience corroborate the beneficial effects of intravesical GAG replenishment therapy in providing symptomatic relief for patients with IC/BPS [9]. Several small-cohort clinical trials have found that bladder pain scores decreased and quality of life improved significantly after intravesical hyaluronic acid or chondroitin sulfate instillation [19,20]. A mixture of heparin and alkalinized lidocaine facilitated the penetration of the lidocaine and provided therapeutic relief [21]. A combination of hyaluronic acid and chondroitin may impart additional benefits [22]. However, intravesical GAG replenishment does not provide long-term efficacy. Although regeneration of the urothelium may increase after intravesical hyaluronic acid instillation, the underlying pathophysiology of chronic inflammation might not be adequately resolved after short-term GAG replenishment [23]. Therefore, repeated intravesical instillation of GAG replenishment is recommended by the American Urological Association (AUA) guidelines [10]. To obtain durable efficacy, repeated intravesical instillation of glycosaminoglycan replenishment or experimental treatments aimed at resolving chronic inflammation to improve urothelial barrier function, such as intravesical botulinum toxin A (BoNT-A) injections, low-energy shock wave (LESW) bladder therapy, and intravesical platelet-rich plasma (PRP) injections, might be necessary [24,25,26,27].

## 4. Chronic Inflammation and Intravesical Treatment for NHIC

Chronic inflammation of the bladder wall is the fundamental pathophysiology of IC/BPS. Increased expression of the inflammatory marker tryptase in the suburothelium has been associated with the increased expression of the apoptotic marker terminal deoxynucleotidyl transferase, decreased expression of the proliferation marker Ki-67, and decreased expression of the adhesive proteins E-cadherin and zonula occludens-1 [23]. A significant increase in urothelial cell apoptosis, as indicated by the high expression of Bad and Bax, in IC/BPS is associated with the increased expression of tumor necrosis factor alpha (TNF-α) [28]. With inflammation-related urothelial dysfunction, there are fewer mature urothelial apical cells marked by CK20 expression in patients with HIC or high-grade glomerulations [29]. The urothelial progenitor cell marker Shh was also significantly decreased in patients with HIC.

Chronic inflammation in IC/BPS may be induced by toxins, bacterial infection, surgical trauma, autoimmune reactions, or systemic disorders [30]. Increased mast cell activity may contribute to IC/BPS through the overexpression of tryptase and increased urinary nerve growth factor levels [31]. However, no recent studies have confirmed mast cell activation as the main source of inflammation in IC/BPS. Urinary biomarker studies have reported elevated inflammatory biomarkers in IC/BPS, which increased with the severity of the bladder condition, such as a smaller maximal bladder capacity or higher-grade glomerulation [13]. The increased expression of serum pro-inflammatory cytokines (interleukin [IL]-1β, IL-6, and TNF-α) and chemokines (IL-8) in IC/BPS implies the significant roles of mast cell activation and inflammatory mediators in the pathogenesis of IC/BPS [32]. Furthermore, increases in sensory receptor expression, nerve hyperplasia, systemic inflammatory reactions, and stress may also contribute to IC/BPS [33].

The medical treatments used to treat chronic inflammation in patients with IC/BPS frequently include non-steroidal anti-inflammatory drugs (NSAIDs), pain killers, COX-2 inhibitors, and β-3 adrenoceptor agonists. However, most of them cannot effectively alleviate pain symptoms [18]. Oral pentosane polysulfate can provide barrier protection for a defective urothelium and improve inflammation and bladder pain in a proportion of patients [34]. Cyclosporin and antihistamines have all been evaluated in small cohorts, and the results are controversial [35,36]. Intravesical dimethyl sulfoxide has been approved by the U.S. Food and Drug Administration and has been deemed effective in reducing bladder pain symptoms without reducing functional bladder capacity [37]. Recent studies have demonstrated the effective use of intravesical injections of botulinum toxin A (BoNT-A), PRP, and high-concentration glucose water in eliminating or reducing bladder pain [38,39,40]. Bladder therapy with suprapubic LESWs can decrease chronic inflammation, improve urothelial regeneration, and alleviate bladder pain in patients with IC/BPS [25,29].

Intravesical BoNT-A injection was first used in the treatment of neurogenic and idiopathic overactive bladders [38]. The efficacy of repeat BoNT-A injections with hydrodistension in improving bladder pain and maximal bladder capacity has previously been demonstrated [41]. The therapeutic effects of BoNT-A injections for IC/BPS were found to be superior to those of a placebo with some durability [42]. In a randomized placebo control trial, at week 8, a significantly greater reduction in pain was observed in the BoNT-A group compared to the control group (−2.6 ± 2.8 vs. −0.9 ± 2.2, *p* = 0.021). Cystometric bladder capacity also increased significantly in the BoNT-A group. The overall success rates were 63% in the BoNT-A group versus 15% in the control group (*p* = 0.028) [42]. Significantly better success rates were noted in patients receiving four repeated injections and three injections compared to a single injection [41]. Repeated intravesical BoNT-A injections can reduce mast cell activity and apoptotic signaling molecule levels, thereby promoting the expression of Ki-67 and E-cadherin [43]. Therefore, intravesical BoNT-A injection is recommended in the clinical guidelines and regarded a standard fourth-line treatment for IC/BPS [8]. Adverse events associated with BoNT-A injection include difficulty urinating, a large post-void residual volume, and subsequent urinary tract infections [44]. Therefore, wide application of this effective treatment has been limited. To reduce the rate and severity of adverse events with BoNT-A, injection at the trigone and the ladder base has been proposed, with a similar therapeutic efficacy [44].

PRP is rich in several kinds of growth factors that promote the resolution of inflammation and facilitate wound healing. PRP has previously been used in many medical applications as regenerative medicine therapy [45]. The use of PRP in the treatment of IC/BPS was first reported in 2019. IC symptom score, functional bladder capacity, and global response assessment were all improved after four consecutive PRP injections. Preliminary studies have demonstrated that multiple intravesical PRP injections could improve symptoms in 70% of IC/BPS patients [46]. Another clinical study on PRP also found improvements in all of the variables measured, including the interstitial cystitis symptom index, the interstitial cystitis problem index, the visual analog scale for pain, functional bladder capacity, frequency, and nocturia. The success rate improved from 45% after the first PRP injection, persisted up to 70% at the primary endpoint, and remained at 67.5% at 3 months after the fourth PRP injection [46]. Increased urothelial cell proliferation and cytoskeleton and barrier function protein expression after repeat PRP injections suggested improved urothelium health in patients with IC/BPS [27]. An ultrastructural study of the urothelial cells from IC/BPS bladders also showed improved urothelial alignment and tight junctions [47]. Adding autologous emulsified fat (Nanofat) to PRP was reported to have very satisfactory outcomes (in terms of bladder pain and IC symptoms) in a small cohort of patients with IC/BPS [48]. There have been few comparative studies on different novel bladder therapies for IC/BPS. One recent study comparing the clinical efficacy of PRP and BoNT-A for IC/BPS revealed no significant difference in symptom improvements or GRA at 6 months. However, only half of the patients in either group had satisfactory treatment outcomes. Patients receiving PRP injections had fewer adverse events, such as dysuria or urinary tract infections, than those receiving BoNT-A injections [46]. 

Local treatment with LESWs has shown demonstrated efficacy in reducing inflammation, improving blood perfusion, and facilitating tissue regeneration. It has been widely applied in the treatment of skeletomuscular disorders and chronic fasciitis [49]. LESWs were recently used to treat male erectile dysfunction and overactive bladder syndrome [50]. The suprapubic application of LESWs to IC/BPS was also effective in improving urothelial regeneration and reducing inflammation. Patients who underwent effective LESW therapy reported a greater pain reduction than those who underwent the placebo treatment [51]. At 12 weeks after LESW therapy, the improvement in the patients with a VAS score ≥ 3 was 57.1% vs. 19.0% (LESWs vs. placebo; *p* = 0.011) and was associated with an improvement in urination frequency. Similar results were also reported by a different study group: a significant improvement in the ICSI and daily frequency in three-day voiding diaries and an increase in average voided volume. However, no significant differences in the urodynamic parameters were noted [52]. A pilot study of using LESWs with intravesical BoNT-A instillation detected cleaved SNAP25 in the suburothelium of IC/BPS bladders, indicating that LESWs could increase urothelial permeability and facilitate the penetration of BoNT-A molecules across the urothelial barrier [26].

## 5. Autoimmune Reactions and Treatment for NHIC

In the early 19th century, Drs. Physick and Parsons [53] first described the chronic inflammatory condition in IC/BPS, which has long been considered an important pathogenic factor of IC/BPS. The widespread infiltration of mast cells, in addition to lymphocytes and plasma cells, into the bladder submucosa and detrusor muscle is also an important characteristic of IC/BPS [54,55,56].

Mast cells are known to release histamine during an immune or allergic response [57], and increased histamine levels have consistently been observed in bladders and urine samples from patients with IC/BPS [56,58]. A recent study revealed increased expression of histamine receptors in IC/BPS bladders, and approximately 63% (38/60) of patients with IC/BPS responded to antihistamine treatment [36]. IC/BPS has long been considered an autoimmune disease. An early study found that 94% of patients with IC/BPS were positive for serum antitissue antibodies. Antinuclear antibodies were also found in 85% of sera at titers of 1:10 or higher [59]. However, bladder-tissue-specific antibodies were not found in these patients, implying that the autoantibodies may have been non-organ-specific [59]. Patients with systemic lupus erythematosus may also have bladder inflammation, which is known as lupus cystitis [60]. Several studies using different laboratory assessments have observed the upregulation of inflammatory cytokines and increased IL-6, IL-10, and TNF-α expression in IC/BPS bladders. The dysregulated expression of inflammatory cytokines in the bladder has a significant impact on pathogenic mechanisms [61]. However, the key mechanism of bladder immune dysregulation and inflammation remains unclear.

Akiyama et al. [62] conducted a genome-wide association study of patients with HIC and found that a genetic variant, rs1794275, located in the major histocompatibility complex (MHC) region (chromosome 6p21.3) was associated with HIC. Fine-mapping of the human leukocyte antigen (HLA) showed amino acid variants of the HLA-DQβ1 and HLA-DPβ1 chains. The variants in HLA-DQβ1 are located together at the peptide-binding groove, indicating their functional importance for antigen presentation. Akiyama et al. showed a possible association between HIC and class II MHC molecule antigen presentation and provided evidence of HIC being a possible autoimmune disorder. A study that performed RNA sequencing and immunochemical staining to characterize the bladders of patients with HIC found that T helper (Th) 1/17-polarized immune responses and the prominent overexpression of interferon gamma [63] Th 17 cells are key regulators of immune responses that produce pro-inflammatory cytokines, including IL-17A, IL-17F, and IL-22. The dysregulation of Th 17 cells is considered an important pathogenic mechanism of autoimmune diseases. Immune disorders involving the bladder may be involved in the pathogenic mechanism in patients with IC/BPS, but their significance may vary highly between different phenotypes of IC/BPS. Inflammatory cell infiltration and aberrant cytokine expression feature in both HIC and NHIC bladders, but evidence shows that the inflammation in HIC bladders is more significant and may result from aberrant autoimmunity.

Regarding aberrant autoimmunity in patients with IC/BPS, targeted treatment for modifying bladder immunity has been investigated in many trials and applied in clinical practice [10]. Because of increased mast cell activity and consequently increased urinary histamine levels [64], the pathogenesis of IC/BPS may involve an allergic reaction. Antihistamines have been used to treat patients with IC/BPS since the 1950s, with hydroxyzine being the most widely used antihistamine for IC/BPS [65,66]. Animal studies revealed that hydroxyzine can inhibit mast cell activity [67], providing evidence supporting the use of hydroxyzine in patients with IC/BPS. Cyclosporine is a widely used immunosuppressive drug. The first clinical trial investigating the use of cyclosporine to treat patients with severe IC/BPS was performed in 1996 [68]. The patients in this clinical trial showed significant improvements in urinary frequency, voided volume, and bladder pain after 3–6 months of treatment. However, most of the patients experienced a symptom relapse after treatment cessation. A randomized controlled study that compared the clinical efficacy of cyclosporine and pentosan polysulfate found that patients treated with cyclosporine showed a greater reduction in urinary frequency and a higher subjective response rate, as measured using the global response assessment [69]. In addition, patients treated with cyclosporine had significantly decreased levels of urinary epidermal growth factor [70].

Tacrolimus is also a widely used immunosuppressant. In a pilot study that used intravesical tacrolimus installation to treat 24 patients with IC/BPS, 56% (13/24) of the patients responded to tacrolimus installation [71]. Monoclonal antibody targeting of TNF-α has been widely used to treat patients with autoimmune diseases [72]. Adalimumab was the first TNF-α-blocking agent used to treat patients with IC/BPS. However, a randomized, double-blind, placebo-controlled trial showed that the reduction in bladder pain in the adalimumab group was not superior to that in the placebo group [73]. Certolizumab pegol, a novel anti-TNF-α agent, was investigated in a randomized, double-blind, placebo-controlled trial [74]. The patients treated with certolizumab pegol showed significant improvements in bladder pain and urinary frequency than those treated with the placebo. Furthermore, the rate of adverse events did not differ significantly between the certolizumab pegol and the placebo groups. Of note, the therapeutic effect of certolizumab pegol was not superior to that of placebo until 18 weeks after the study; thus, the therapeutic effects of anti-TNF-α agents may take longer to manifest.

Prednisolone, another widely used immunosuppressant, can suppress bladder inflammation and is the main treatment for lupus cystitis [75,76]. A case series that evaluated using low-dose prednisolone (10 mg once a day) administered for 1–3 months to treat seven patients with NHIC who had experienced symptom flare-ups after previous treatments showed significant improvements in bladder pain and urinary frequency [77]. Akiyama et al. [78] recently reported a long-term effect of low-dose prednisolone in patients with HIC. The response rate at 12 months was 64.5%, and the mean maintenance dose was 3.0 mg. Both prednisolone studies found no patients who discontinued treatment due to adverse events. However, while prednisolone is a feasible treatment for severe IC/BPS, patients need to be regularly followed up to adjust the dose.

## 6. Pelvic Floor Dysfunction and Psychological Factors

A high proportion of patients with IC/BPS exhibit pain in the pelvic floor, urethra, and perineum, in addition to characteristic bladder symptoms [79]. The AUA guidelines recommend classifying these patients into another phenotype of IC/BPS [10]. More wide-spreading pelvic pain is usually associated with more severe IC bladder symptoms and a lower quality of life [80]. Pelvic pain in male patients with IC/BPS is also common, and these patients may be diagnosed with chronic prostatitis/chronic pelvic pain syndrome [10,81]. In female patients, physiotherapy at the trigger points can effectively reduce pelvic pain [82].

In addition to pelvic pain, patients with IC/BPS also present with many functional somatic syndromes, such as chronic fatigue syndrome, irritable bowel syndrome, tension-type headaches, migraine, temporomandibular disorders, myofascial pain syndrome, regional soft-tissue pain syndrome, periodic limb movements in sleep, multiple chemical sensitivity, primary dysmenorrhea, female urethral syndrome, post-traumatic stress disorder, and fibromyalgia syndrome. Patients may also have anxiety and depression [83]. These patients may likely have a common pathway in inducing these functional somatic syndromes. This is also reflected in an increase in serum biomarkers, such as C-reactive protein, pro-inflammatory cytokines (IL-1β, IL-6, and TNF-α), and chemokines (IL-8) [30]. Treatment with NSAIDs and other anti-inflammatory agents are usually prescribed but may fail to eradicate these somatic syndromes.

Increased anxiety and depression has been observed in 60% of patients with IC/BPS [84]. Furthermore, the expression of corticotropin-releasing hormone receptors is also elevated in patients with IC/BPS [33]. IC/BPS may result from a bladder insult and form a vicious cycle that enhances the central perception of bladder symptoms, which bidirectionally increases the severity of anxiety and depression [85]. Patients with a high anxiety or depression index should be prescribed anti-anxiety or antidepressant medication. Psychiatric consultation in addition to bladder therapy is usually effective in reducing psychological stress and improving quality of life [86].

## 7. Pathophysiology, Clinical Presentations, and Treatment for HIC

The mainstay clinical symptoms of HIC are severe bladder pain and a contracted bladder [87]. Cystoscopy in the clinic without anesthesia can reveal typical solitary, multiple, or diffuse HIC lesions on the posterior bladder wall. The cystoscopic presentation of HIC lesions is not uniform and may present as one of the following: (A) a dense inflammatory lesion with erosive mucosa; (B) an erythematous patch with radiating vessels; (C) a small scar with erythema and radiating vessels; or (D) a small, denuded mucosal patch and focal thickening [88]. The bladder wall affected by HIC lesions usually presents with thickening in computed tomography or magnetic resonance imaging of the bladder [89]. Urine inflammatory and oxidative stress biomarkers are usually significantly elevated in patients with HIC compared with those with NHIC or controls [90,91].

Recent investigations of the bladder wall beneath urothelial HIC lesions have reported dense inflammatory tissues. The commonly reported histopathological findings in HIC are urothelial denudation, lymphoplasmatic cell infiltration, eosinophilic cell infiltration, lamina propria hemorrhage, and suburothelial granulation [92]. The histopathological changes in HIC and NHIC with grade 3 glomerulation are more severe that those in NHIC bladders with low-grade glomerulation [30]. In HIC, lymphoid follicle aggregation in 40% of cases and high-grade B-cell infiltration also indicate immune-mediated inflammation, and these inflammatory reactions in HIC are distinct from those in NHIC [93]. The histopathological changes in HIC bladders are compatible with the diffuse bladder wall thickness noted in HIC or NHIC with high-grade glomerulation [16]. Diffuse or focal thickening of the bladder wall has been noted in all HIC bladders, all patients with European Society for the Study of Interstitial Cystitis type 3 bladders, and most patients with a maximal bladder capacity < 760 mL and grade 2 or 3 glomerulation [29].

Our recent analysis of the pathophysiology of HIC using polymerase chain reaction and in situ hybridization detected Epstein–Barr virus (EBV) in 87.5% of the bladder tissue from patients with HIC and 17.4% of the patients with NHIC with high-grade glomerulation on cystoscopic hydrodistension [94]. Interestingly, both latent and lytic EBV infections were detected in the bladders of patients with HIC, indicating that chronic inflammation in HIC bladders is caused by the persistence or reactivation of EBV infection [94]. This finding of EBV infection in HIC and NHIC is important because IC/BPS symptoms may flare up over time, and antiviral agents can be used to treat patients who are refractory to conventional therapy. Latent EBV infection in the B cells of HIC bladders also induces the increased expression of brain-derived neurotrophic factors, which could be the cause of nerve hyperplasia and bladder hypersensitivity in patients with IC/BPS [94].

The primary treatment for HIC should be the fulguration of HIC lesions, either by electrocauterization or laser ablation [9,10]. Partial cystectomy with or without bladder augmentation is necessary in patients with HIC and a severely contracted bladder [11,95]. Although invasive surgery can quickly alleviate bladder symptoms and functional bladder capacity, the long-term risks should be considered, and patients should be educated on the procedure. Antiviral medications, such as oral valacyclovir treatment, can effectively relieve IC symptoms and reduce urinary inflammatory biomarker levels, which shows that antiviral therapy can efficiently treat HIC with EBV infection [96]. However, the treatment duration and the biomarkers for determining the duration of antiviral treatment have not been established. A recent clinical trial on the intravesical instillation of interferons also demonstrated an effective reduction in bladder pain, further highlighting the efficacy of an antiviral treatment strategy for HIC [97].

## 8. Multimodal Active Therapy Based on the Pathophysiology of IC/BPS

Although BoNT-A, PRP, and LESWs have been shown to effectively reduce inflammation, improve urothelial regeneration, and improve IC symptoms in IC/BPS bladders, the relevant clinical trials are few. A randomized comparative trial is still lacking [98]. A comprehensive search of the literature for randomized control trials of different intravesical therapies for IC/BPS showed statistically significant improvements in IC symptoms compared to the controls or placebos [99]. Nevertheless, because treatment of IC/BPS starts with behavioral modifications and progresses through medical treatment and intravesical instillation to minimally invasive intravesical injections, patients who receive novel bladder therapy usually have failed traditional therapy. Therefore, there has been no comparison of the safety and efficacy between the novel and conventional treatments for IC/BPS. Regarding the therapeutic efficacy of different novel bladder therapies, the selection of IC/BPS patients with a certain clinical phenotype for appropriate bladder therapy might determine the treatment outcomes. Therefore, there is no best bladder treatment, only the most suitable treatment for IC/BPS. An effective bladder treatment for IC/BPS is not dependent on the potency of therapy but is based on the correct treatment targeting the underlying pathophysiology of IC/BPS, that is, selecting the right IC/BPS patients for the right bladder treatment. 

Since the pathophysiology of IC/BPS is multifactorial, an algorithm for treatment should be established in individual patients with IC/BPS. Bladder pain symptoms can be used to distinguish between HIC and NHIC. If a patient has severe bladder pain and a reduced bladder capacity and focal or diffuse bladder thickness on computed tomography, outpatient cystoscopy should be performed to search for Hunner’s lesions. Once Hunner’s lesions are detected, fulguration by electrocauterization or laser ablation should be performed and antiviral agents administered, followed by repeat fulguration or subsequent invasive surgery, depending on the clinical evaluation regarding the removal of the severely inflammatory bladder wall.

**Figure 3:**
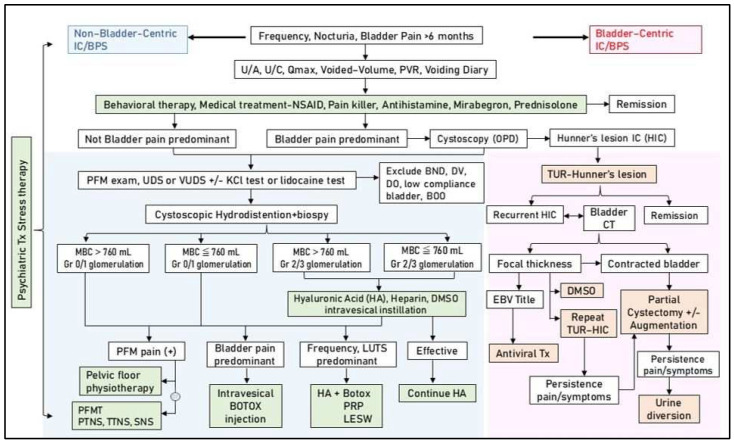
Multimodal therapy and strategy for the treatment of interstitial cystitis/bladder pain syndrome (IC/BPS). Abbreviations: U/A: urinalysis, U/C: urine culture, Qmax: maximum flow rate, PVR: post-void residual, NSAID: non-steroidal anti-inflammatory agent, PFM: pelvic floor muscle, UDS: urodynamic study, VUDS: videourodynamic study, KCl: potassium chloride, BND: bladder neck dysfunction, DV: dysfunctional voiding, DO: detrusor overactivity, TUR: transurethral resection, CT: computed tomography, HIC: Hunner’s interstitial cystitis, MBC: maximum bladder capacity, Gr: grade, EBV: Epstein–Barr virus, LUTS: lower urinary tract symptoms, PFMT: pelvic floor muscle training, PTNS: percutaneous tibial nerve stimulation, TTNS: transcutaneous tibial nerve stimulation, SNS: sacral nerve stimulation, PRP: platelet-rich plasma, LESW: low-energy shock wave.

A detailed historical review and physical examination should be performed for patients with NHIC and persistent IC symptoms. Patients may be treated with oral anti-inflammatory medication or analgesics for frequent urination and bladder pain. If bladder symptoms persist, we recommend performing cystoscopic hydrodistension under anesthesia, and the maximal bladder capacity under an intravesical pressure of 80 cm H_2_O and the glomerulation grade should be recorded. For patients with significant bladder pain and high-grade glomerulation, intravesical BoNT-A injection every 6 months or suprapubic LESW bladder treatment, followed by intravesical instillation of GAG supplementation, is recommended. For patients with frequency-predominant symptoms, monthly intravesical PRP injections for four months followed by intravesical instillation of GAG supplements are advised. If tenderness points in the pelvic floor muscles are observed, regular pelvic floor physiotherapy should be performed in combination with bladder therapy. LESW treatment for pelvic floor muscle pain is also recommended in addition to physiotherapy. All patients should also be evaluated in terms of their anxiety or depression status. If their anxiety or depression index is high, then anti-anxiety or antidepressant medication should be prescribed. If feasible, psychiatric consultation during active bladder therapy should be performed to reduce psychological stress and IC bladder sensitivity. Figure 3 shows the proposed algorithm of multimodal therapy and strategy for the treatment of IC/BPS.

## 9. Conclusions

The pathophysiology of IC/BPS includes chronic inflammation, viral infection, urothelial dysfunction, sensory nerve hyperplasia, lymphoplasmatic cell infiltration, chronic lymphoid follicle aggregation, bladder wall thickening, central nervous system sensitization, extravesical inflammation, and psychological stress. Treatment of IC/BPS should distinguish between different clinical phenotypes, the underlying pathophysiology causing clinical symptoms, and visual exploration of bladder lesions. Furthermore, the treatment strategy should be individualized to adequately address the needs of patients with IC/BPS. With the literature basis of this review, we propose using multimodal active therapy to provide effective personalized treatment of appropriately selected patients with IC/BPS.
